# Feasibility and acceptability of insecticide treated plastic sheeting (ITPS) for vector control in Papua New Guinea

**DOI:** 10.1186/1475-2875-11-S1-P110

**Published:** 2012-10-15

**Authors:** Justin Pulford, Anthony Tandrapah, Jo-An Atkinson, Brown Kaupa, Tanya Russell, Manuel W Hetzel

**Affiliations:** 1 Papua New Guinea Institute of Medical Research, Goroka, EHP 441, Papua New Guinea; 2 The University of Queensland, School of Population Health, Herston, QLD 4006, Australia; 3 James Cook University, Faculty of Medicine, Health and Molecular Sciences, Cairns, Australia

## Background

This study assessed the feasibility and acceptability of utilising insecticide treated plastic sheeting (ITPS) as a malaria control intervention in Papua New Guinea (PNG).

## Method

Zero Vector^®^ ITPS was installed in 40 homes across four study sites representing a cross section of malaria transmission risk and housing style. Structured questionnaires were completed at the time of ITPS installation (n=40) and at four weeks post installation (n=40) with the household head. Similarly, focus group discussions (FGDs) with the male and/or female household heads were completed at installation (n=5) and four week follow-up (n=4).

## Results

ZeroVector^®^ ITPS was successfully installed in a range of homes employing traditional and/or modern building materials in PNG. The ITPS installations remained intact over the course of the four week trial period and were highly acceptable to both male and female household heads. No dissatisfaction with the ITPS product was reported at four week follow-up; however, the installation process was time consuming, participants reported a reduction in mosquito net use following ITPS installation and many participants expressed concern about the longevity of ITPS over the longer term.

## Conclusion

ZeroVector^®^ ITPS installation is feasible and highly acceptable in a diverse range of PNG contexts and is likely to be favourably received as a vector control intervention if accessible en masse. A longer-term evaluation is required before firm policy or public health decisions can be made regarding the potential application of ITPS in the national malaria control program. The positive study findings suggest a longer-term evaluation of this promising malaria control intervention warrants consideration.

